# Prevalence of underweight, overweight and obesity and their associated risk factors in Nepalese adults: Data from a Nationwide Survey, 2016

**DOI:** 10.1371/journal.pone.0205912

**Published:** 2018-11-06

**Authors:** Lal B. Rawal, Kie Kanda, Rashidul Alam Mahumud, Deepak Joshi, Suresh Mehata, Nipun Shrestha, Prakash Poudel, Surendra Karki, Andre Renzaho

**Affiliations:** 1 Western Sydney University, Sydney, Australia; 2 JICA, Accra, Ghana; 3 University of Southern Queensland, Toowoomba, Australia; 4 Save the Children, Kathmandu, Nepal; 5 Ipas Nepal, Kathmandu, Nepal; 6 Victoria University, Melbourne, Australia; 7 Australian Red Cross Blood Service, Sydney, Australia; Weill Cornell Medical College Qatar, QATAR

## Abstract

**Introduction:**

Over the past few decades, the total population of Nepal has increased substantially with rapid urbanization, changing lifestyle and disease patterns. There is anecdotal evidence that non-communicable diseases (NCDs) and associated risk factors are becoming key public health challenges. Using nationally representative survey data, we estimated the prevalence of underweight, overweight and obesity among Nepalese adults and explored socio-demographic factors associated with these conditions.

**Materials and methods:**

We used the Nepal Demographic Health Survey 2016 data. Sample selection was based on stratified two-stage cluster sampling in rural areas and three stages in urban areas. Weight and height were measured in all adult women and men. Body mass index (BMI) was calculated using Asian specific BMI cut-points.

**Results:**

A total of 13,542 adults aged 18 years and above (women 58.19%) had their weight and height measured. The mean (±SD) age was 40.63±16.82 years (men 42.75±17.27, women 39.15±16.34); 41.13% had no formal education and 60.97% lived in urban areas. Overall, 17.27% (95% CI: 16.64–17.91) were underweight; 31.16% (95% CI: 30.38–31.94) overweight/obese. The prevalence of both underweight (women 18.30% and men 15.83%, p<0.001) and overweight/obesity (women 32.87% and men 28.77%, p<0.001) was higher among women. The older adults (≥65 years) (aOR: 2.40, 95% CI: 1.92–2.99, p<0.001) and the adults of poorest wealth quintile (aOR: 2.05, 95% CI: 1.62–2.59, p<0.001) were more likely to be underweight. The younger age adults (36–45 years) (aOR: 3.05, 95% CI: 2.61–3.57, p<0.001) and women (aOR: 1.53, 95% CI 1.39–1.68, p<0.001) were more likely to be overweight or obese. Also, all adults were twice likely to overweight/obese (p<0.001). No significant difference was observed for overweight/obesity by ecological regions and place of residence (urban vs. rural).

**Conclusion:**

These findings confirm co-existence of double burden of underweight and overweight/obesity among Nepalese adults. These conditions are associated with increased risk of developing NCDs. Therefore, effective public health intervention approaches emphasizing improved primary health care systems for NCDs prevention and care and using multi-sectoral approach, is essential.

## Introduction

Chronic non-communicable diseases (NCDs) are the major causes of disease burden and mortality in the Asia and Pacific region, claiming 55% of total life in the South East Asia region each year [1–3]. Further, it is projected that NCDs in Asia will account for up to 80% of all deaths and 40% of all morbidity by 2030, if no appropriate actions are taken [1]. The World Health Organization (WHO) estimated that South Asian countries recorded 21% increase in total mortality in a 10-year time frame (2005–2015), which was the highest increase worldwide [4] and the NCD related deaths increased the most in the WHO South-East Asia Region [1]. The increase in NCDs burden not only presents a major threat to already deteriorating health situation of the general population, but also negatively affects the overall socio-economic development of the countries [5, 6]. Such a pattern ultimately poses a threat to achieving Sustainable Development Goals (SDG), by 2030 [7]. The third SDG targets a one-third reduction in premature mortality due to NCDs. Some of the key drivers of NCDs in low and middle-income countries (LMICs) include the nutrition transition and associated overweight and obesity, rapid urbanization, changing lifestyles, advance health care and ageing population [1].

Nepal, with a population of almost 29 million in 2016 [8], has been facing increasing burden of chronic NCDs over the last 20 years [9, 10]. The evidence base on NCDs from the population-based data is scarce, however results from several small-scale studies conducted in community and hospital settings suggest that the number of people with NCDs including cardiovascular disease (CVD), diabetes, chronic obstructive pulmonary disease (COPD) and cancer is increasing [10–12]. According to the WHO, approximately 60% of total deaths (aged between 30 and 70 years) are attributable to NCDs and NCD-related conditions in Nepal [13]. Nepal has higher age-standardized death rates and disability-adjusted life years from NCDs than communicable diseases [7]. The common modifiable risk factors for NCDs include tobacco use, harmful use of alcohol, inadequate intake of fruits and vegetables, high salt and trans-fat consumption, and physical inactivity [1, 2]. These risk factors are highly prevalent among the Nepalese adults [14]. According to the WHO STEP wise approach to surveillance (STEPS) survey 2013, 17.7% of the Nepalese adults were overweight, 4% obese, 12.3% currently consuming alcohol, 17.8% current tobacco users and only 1.1% having sufficient fruits and vegetables intake [14]. Further, the survey reported almost 41% of adults had at least one NCD risk factor, 30.9% had 2 risk factors, 18.7% had 3 risk factors and 9.0% had 4 or more NCD risk factors [14]. Nepal’s total population increased substantially from 15 million in 1981 to almost double in 2016. The life expectancy at birth also increased from 49.5 to 68 years during the same period [8, 15] and the patterns of diseases and associated risk factors have changed with predominance of NCDs and related conditions [14]

Despite efforts in improving nutritional status of the Nepalese people, underweight, overweight and obesity still remain serious public health challenges [14, 16]. These conditions can lead to development of NCDs, risking people to immature death and disability [17, 18]. While some studies have sought to examine NCDs and associated factors, they have been regionally focused and limited to hospital/ community settings [10–12], hence limiting their external validity. Evidence from nationally representative samples are urgently needed. The aim of this study was to estimate prevalence of underweight, overweight and obesity and its’ associated socio-demographic and behavioural factors among the adult population in Nepal.

## Materials and methods

### Study design and sampling

We performed secondary analyses of data available from Nepal Demographic and Health Survey (NDHS), 2016. A detail methodology of NDHS has been presented elsewhere [19]. In brief, NDHS is a cross-sectional nationally representative survey conducted between 19 June 2016 and 31 January 2017 and was a collaboration between New ERA Nepal, Ministry of Health (MOH), Nepal, ICF International USA and USAID. Participants of this survey were selected using stratified two stage cluster sampling in rural areas and three stages cluster sampling in urban areas. In rural areas, wards were selected as primary sampling units (PSUs), and households were selected from the sample PSUs. In urban areas, wards were selected as PSUs, one enumeration area (EA) was selected from each PSU, and then households were selected from the sample EAs. Firstly, 383 primary sampling units (wards) were selected with probability proportional to ward size. Subsequently, a fixed number of 30 households per cluster were selected with an equal probability systematic selection from the households listing. Altogether, interviews were completed in 11,040 households. This subsample analyses included 13,542 adults aged 18 years and above with Males 5,662 (underweight 896 (15.83%), normal weight 3,136 (55.40%) and overweight or obese 1,629 (28.77%)) and Females 7881 (underweight 1,442 (18.30%), normal weight 3,848 (48.83%) and overweight or obese 2,591 (32.87%). After exclusion of non-responders and participants with missing data for anthropometric measurements, we included 13,542 adults for this subsample analysis.

### Outcome measurement

In NDHS, 2016, the weight and height of the participants were measured at the participant’s home by two female trained field research staff. Weight was measured once with light clothing on and without shoes by digital weighing scales placed on a flat surface. Height was measured once using a standard clinical height measuring scale with participant standing without shoes. The participants who could not stand had their height measured in a lying position. Body mass index (BMI) was calculated as weight (kg)/height (m^2^). Using Asian specific BMI cut-offs underweight was defined as <18.5 kg/m^2^, normal weight as 18.5–22.99 kg/m^2^, overweight as 23–27.49 kg/m^2^ and obese ≥27.5 kg/m^2^ [20].

### Explanatory variables

The study variables were selected a-priori based on prior studies, a review of the relevant published studies, and the available information in the DHS datasets, with a consideration of potential confounders. Individual-level factors such as age, sex, educational status, marital status, and nutritional status were collected by questionnaire which was administered during a face to face interview. Community-level factors, such as household wealth status and place of residence (urban or rural), administrative divisions, ecological zone (Mountain, Hill or Terai) were also considered in the study. Province was categorized into seven administrative divisions, according to the current administrative structure of Nepal. The DHS applied an asset-based approach to estimate household wealth status, and has been described previously [21]. Each variable (asset) was dichotomized as 1 if present and 0 if not, and the wealth index was constructed using principal component analysis (PCA). Weights were determined by factor scores derived from the first principal component in the PCA. The constructed wealth index values were then assigned to each individual based on common variables.

### Statistical analyses

Data were analyzed using Stata/SE 13.0 (StataCorp, College Station, TX, USA). In the descriptive analyses, the characteristics of the study participants are presented in the form of frequency (n), the percentages (%) with 95% confidence interval (CI) or rmean with standard deviation. Univariate and multivariable logistc regression models were used to examine the relationship betweem the participants’ nutrional status (underweight and overweight/obesity compared to normal weight) and socio-demographic and economic variables, adjusted with sampling weight and clustering effect. The variables having p-value ≤0.05 in the bivariate analysis were entered into multinomial logistic regression models to control the confounding effect. The goodness of fit model was employed using the Hosmer and Lemeshow statistic [22]. Variance Inflation Factor (VIF) test was done to determine whether multicollinearity was present or not [23]. For all the tests conducted in the study, a P-value of 0.05 or below was considered as the statistically significant level.

### Ethical consideration

The ethics approval for NDHS, 2016 was obtained from the Ethical Review Board of Nepal Health Research Council and ICF Institutional Review Board. The DHS data are publicly accessible and were made available to us upon request by Measure DHS.

## Results

### Socio-demographic characteristics of the study participants

**Fig 1:**
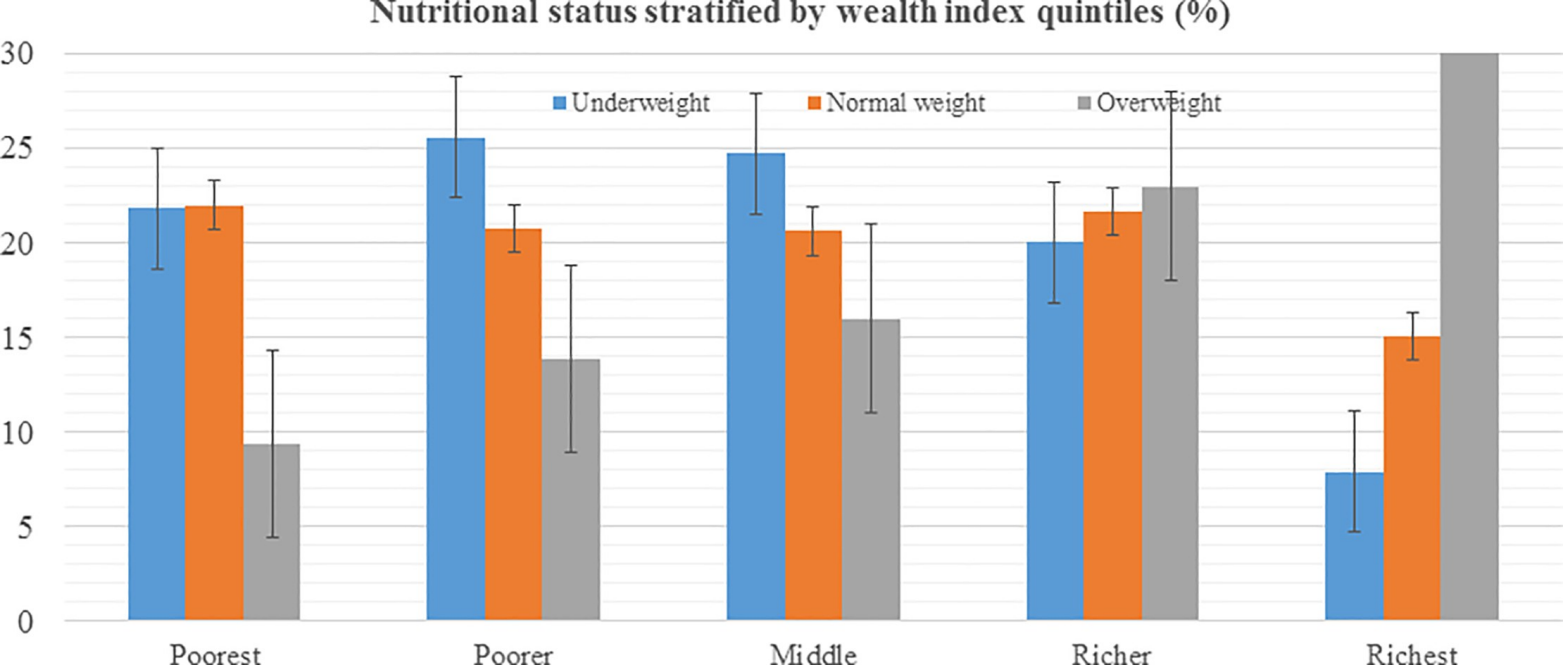
Nutritional status of the participants stratified by wealth index quintile.

**Fig 2:**
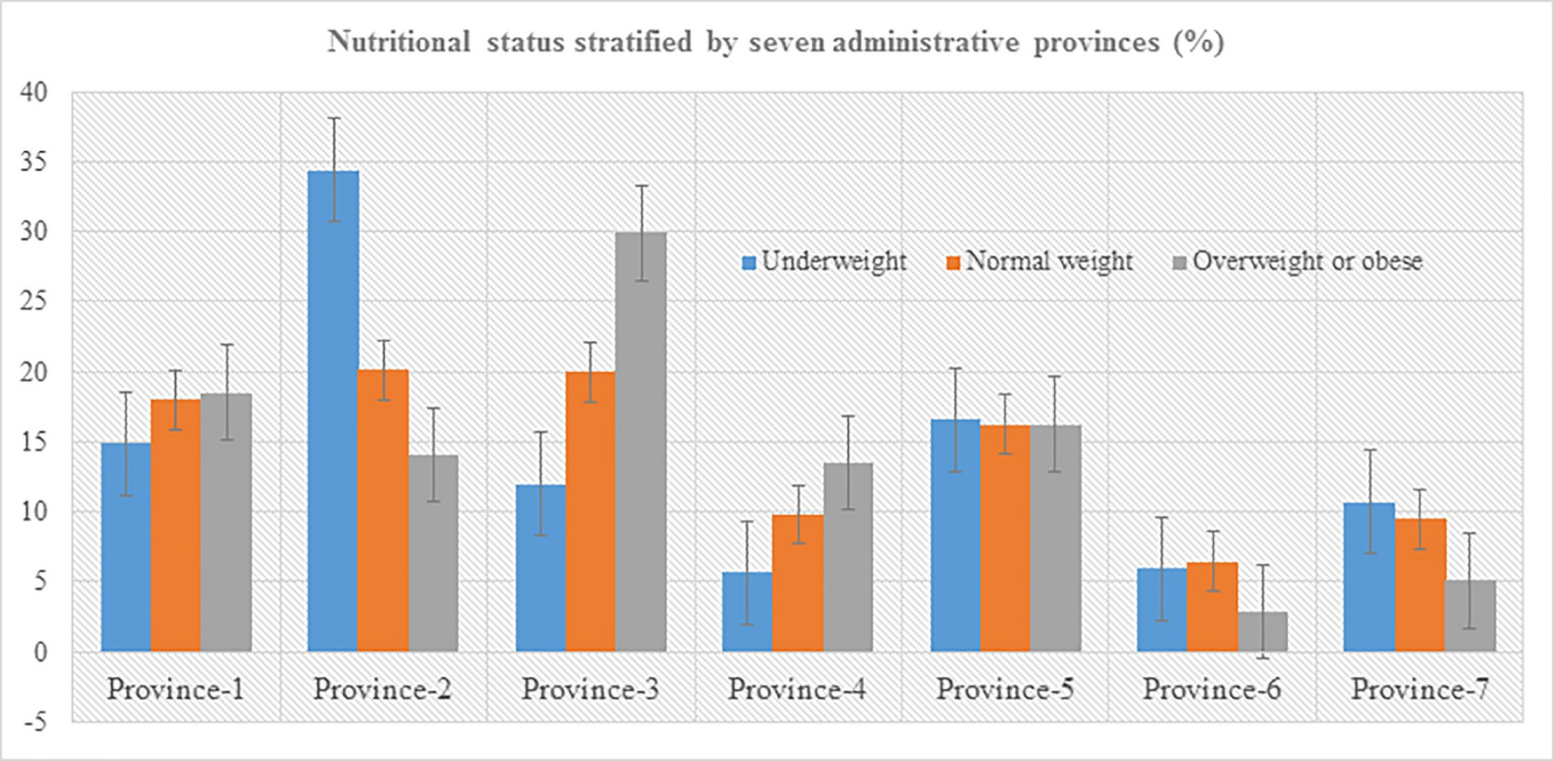
Nutritional status of the participants stratified by seven administrative provinces.

**Table 1** describes socio-demographic characteristics of the total 13,542 study participants included in this study. The mean (± SD) age was 40.63 ± 16.82 years, with males 42.75 ± 17.27 and females 39.15 ± 16.34 years. The proportion of female participants was 58.19%, over a third (41.13%) did not attain formal education and 60.97% lived in urban areas. In terms of wealth status of the participants, there was not much variation among the categories from poorest to richest quintiles, each in an average representing around 20%. Overall, 2,338 adults (17.27%, 95% CI: 16.64–17.91) were underweight, and 4,219 (31.16%, 95% CI: 30.38 31.94) were overweight or obese. By sex, the prevalence of overweight or obesity among women was slightly higher (32.87%) than men (28.77%) and this pattern was similar for underweight as well (women 18.30% and men 15.83%). The nutritional status of the participants stratified by the wealth index is presented in **Fig 1** and by seven provincial administrative divisions in **Fig 2**. Large disparities in terms of nutritional status were observed when stratified by both wealth index and administrative divisions. The patterns of overweight or obesity increased by wealth index from poorest as low to richest adults the highest.

**Table 1:** Background characteristics of study participants.

Variables		n (%)	95% CI
**Sex**			
	Male	5661 (41.81)	(40.98–42.64)
	Female	7881 (58.19)	(57.36–59.02)
**Age group (years)**			
	18–25	3126 (23.08)	(22.38–23.80)
	26–35	3107 (22.94)	(22.24–23.66)
	36–45	2463 (18.19)	(17.55–18.85)
	46–55	1987 (14.68)	(14.09–15.28)
	56–65	1552 (11.46)	(10.94–12.01)
	>65	1307 (9.65)	(9.16–10.16)
**Educational background**			
	No education or preschool	5570 (41.13)	(40.31–41.96)
	Primary education	2294 (16.94)	(16.32–17.58)
	Secondary education	3721 (27.47)	(26.73–28.23)
	Higher education	1957 (14.45)	(13.87–15.05)
**Marital Status**			
	Never married	1539 (11.37)	(10.84–11.91)
	Married	10827 (79.95)	(79.26–80.61)
	Others-widowed or divorced	1176 (8.68)	(8.22–9.17)
**Body mass index**			
	Under weight	2338 (17.27)	(16.64–17.91)
	Normal weight	6985 (51.58)	(50.74–52.42)
	Overweight or Obese	4219 (31.16)	(30.38–31.94)
**Wealth quintile**			
	Poorest	2441 (18.03)	(17.39–18.68)
	Poorer	2633 (19.44)	(18.78–20.12)
	Middle	2691 (19.87)	(19.21–20.55)
	Richer	2948 (21.77)	(21.08–22.47)
	Richest	2829 (20.89)	(20.21–21.58)
**Ecological Zone**			
	Mountain	893 (6.59)	(6.19–7.02)
	Hill	5911 (43.65)	(42.81–44.48)
	Terai	6738 (49.76)	(48.92–50.60)
**Residence**			
	Urban	8256 (60.97)	(60.14–61.78)
	Rural	5286 (39.03)	(38.22–39.86)
**Provinces**			
	Province-1	2385 (17.61)	(16.98–18.26)
	Province-2	2800 (20.68)	(20.00–21.37)
	Province-3	2934 (21.67)	(20.98–22.37)
	Province-4	1386 (10.24)	(9.74–10.76)
	Province-5	2204 (16.28)	(15.67–16.91)
	Province-6	708 (5.23)	(4.86–5.61)
	Province-7	1125 (8.31)	(7.86–8.79)

### Factors associated with underweight

In univariate analyses, being underweight was significantly associated with sex, age, education, wealth, ecological zone, province and place of residence (urban vs. rural) (See **Table 2 and Table 3**). After adjusting for sex, education, wealth, and place of residence, older adults (≥65 years of age) were more than twice as likely (adjusted odds ratio (aOR): 2.40, 95% CI: 1.92–2.99, p<0.001) to be underweight than the younger adults. The female participants were more likely (aOR:1.29, 95% CI 1.16–1.44) to expose to underweight compared to males. Similarly, those who had no education or primary level of education only, were more likely (aOR: 1.40 and aOR 1.27, respectively) to be underweight compared to those with a college or higher education. The poorest quintile adults were over twice more likely (aOR: 2.05, 95% CI: 1.62–2.59, p<0.001) to be underweight compared to the adults in wealthy quintiles. Further, those adults living in Terai (low-land) areas were more likely (aOR: 1.45, 95% CI 1.16–1.82) to be underweight compared to those adults of other ecological zones.

**Table 2:** Association between nutritional status and socio-demographic characteristics of study participants (N = 13,542) *.

Variables		Underweight		Normal weight		Overweight/ obesity		P-value
		n	% (95% CI)	n	% (95% CI)	n	% (95% CI)	
**Sex**								
	Male	896	15.83 (14.67–17.07)	3,136	55.40 (53.89–56.89)	1,629	28.77 (27.14–30.46)	<0.001
	Female	1,442	18.30 (16.94–19.73)	3,848	48.83 (47.31–50.35)	2,591	32.87 (31.03–34.77)	
**Age group**								
	18–25	616	19.70 (17.96–21.57)	1945	62.21 (60.17–64.22)	565	18.08 (16.48–19.80)	<0.01
	26–35	346	11.13 (09.53–12.97)	1571	50.57 (48.39–52.74)	1190	38.3 (35.86–40.80)	
	36–45	282	11.45 (09.91–13.2)	1117	45.35 (42.70–48.03)	1064	43.19 (40.15–46.29)	
	46–55	302	15.19 (13.42–17.15)	957	48.16 (45.66–50.66)	728	36.65 (33.97–39.42)	
	56–65	350	22.55 (20.02–25.3)	792	51.02 (48.09–53.93)	410	26.43 (23.53–29.55)	
	>65	442	33.85 (30.57–37.30)	603	46.13 (43.14–49.16)	262	20.02 (17.34–22.99)	
**Education background**								
	No education or preschool	1344	24.12 (22.57–25.74)	2873	51.57 (49.86–53.28)	1354	24.31 (22.59–26.11)	<0.001
	Primary education	357	15.55 (13.99–17.26)	1189	51.82 (49.42–54.22)	749	32.62 (30.35–34.98)	
	Secondary education	442	11.88 (10.65–13.23)	1922	51.66 (49.48–53.83)	1357	36.46 (34.08–38.91)	
	Higher education	196	9.99 (8.25–12.05)	1001	51.16 (48.43–53.88)	760	38.85 (36.05–41.73)	
**Marital Status**								
	Never married	306	19.86 (17.32–22.67)	1000	64.93 (61.99–67.77)	234	15.2 (13.21–17.44)	<0.01
	Married	1688	15.59 (14.56–16.67)	5437	50.22 (48.86–51.58)	3702	34.19 (32.43–36.00)	
	Others-widowed or divorced	345	29.32 (26.37–32.45)	548	46.6 (43.05–50.18)	283	24.09 (21.09–27.37)	
**Wealth quintile**								
	Poorest (Q1)	510	20.89 (18.61–23.36)	1535	62.89 (60.76–64.98)	396	16.22 (14.55–18.03)	<0.001
	Poorer (Q2)	598	22.71 (20.14–25.51)	1450	55.08 (52.75–57.39)	585	22.21 (19.89–24.71)	
	Middle (Q3)	578	21.46 (19.32–23.78)	1439	53.46 (51.40–55.52)	675	25.07 (22.87–27.41)	
	Richer (Q4)	468	15.89 (14.03–17.94)	1510	51.23 (49.19–53.26)	969	32.89 (30.51–35.36)	
	Richest (Q5)	184	6.51 (5.26–8.05)	1050	37.12 (34.72–39.58)	1595	56.37 (53.67–59.03)	
**Ecological Zone**								
	Mountain	145	16.20 (12.42–20.85)	530	59.37 (56.31–62.36)	218	24.43 (19.24–30.49)	<0.005
	Hill	713	12.06 (10.88–13.34)	3108	52.59 (50.55–54.61)	2090	35.36 (32.89–37.91)	
	Terai	1481	21.98 (20.29–23.75)	3346	49.66 (48.02–51.30)	1911	28.36 (26.20–30.63)	
**Residence**								
	Urban	1232	14.92 (13.44–16.53)	4058	49.15 (47.47–50.83)	2966	35.93 (33.67–38.25)	<0.001
	Rural	1106	20.93 (19.13–22.86)	2927	55.37 (53.60–57.13)	1253	23.70 (21.73–25.79)	
**Provinces**								
	Province-1	348	14.6 (12.04–17.59)	1257	52.72 (49.40–56.02)	779	32.68 (29.05–36.53)	<0.001
	Province-2	804	28.71 (26.40–31.14)	1404	50.14 (47.99–52.29)	592	21.15 (18.71–23.81)	
	Province-3	279	9.52 (07.24–12.42)	1393	47.48 (44.12–50.86)	1262	43.00 (38.98–47.12)	
	Province-4	132	9.51 (07.92–11.37)	685	49.40 (46.89–51.92)	570	41.09 (37.72–44.54)	
	Province-5	387	17.54 (14.93–20.50)	1134	51.45 (48.77–54.13)	683	31.00 (27.18–35.11)	
	Province-6	138	19.52 (16.40–23.06)	450	63.60 (60.65–66.46)	119	16.88 (13.79–20.50)	
	Province-7	250	22.23 (19.10–25.70)	661	58.79 (55.18–62.30)	214	18.98 (14.14–25.01)	

* All analysis was adjusted by weight, P-value was derived by chi-square text

**Table 3:** Adjusted odds ratios for factors associated with underweight compared to normal weight and overweight/ obesity compared to normal weight.

Variables		Model-1: Underweight vs. normal weight			Model-2: Overweight/obesity vs. normal weight		
		OR (95% CI)	P-Value	VIF	OR (95% CI)	P-Value	VIF
**Sex**							
	Male	Ref			Ref		
	Female	1.29 (1.16–1.44)	<0.001	2.68	1.53 (1.39–1.68)	<0.001	2.73
**Age group**							
	18–25	Ref			Ref		
	26–35	0.71 (0.60–0.85)	<0.001	1.92	2.24 (1.95–2.58)	<0.001	1.97
	36–45	0.81 (0.67–0.98)	<0.05	1.96	3.05 (2.61–3.57)	<0.001	2.00
	46–55	0.93 (0.77–1.14)	0.26	2.00	2.84 (2.40–3.37)	<0.001	2.04
	56–65	1.48 (1.21–1.81)	<0.001	2.07	2.01 (1.66–2.45)	<0.001	2.10
	>65	2.40 (1.92–2.99)	<0.001	2.28	1.80 (1.43–2.27)	<0.001	2.32
**Education background**							
	No education or preschool	1.4 (1.12–1.74)	<0.001	4.86	0.66 (0.56–0.78)	<0.001	4.95
	Primary education	1.27 (1.02–1.59)	0.03	2.75	0.98 (0.83–1.15)	0.98	2.79
	Secondary education	1.03 (0.85–1.25)	0.78	3.04	1.05 (0.91–1.21)	0.35	3.09
	Higher education	Ref			Ref		
**Marital Status**							
	Never married	1.67 (1.40–2.00)	<0.001	1.44	0.45 (0.37–0.54)	<0.001	1.51
	Married	Ref			Ref		
	Others-widowed or divorced	1.22 (1.03–1.44)	<0.05	1.42	0.96 (0.81–1.15)	0.69	1.43
**Wealth quintile**							
	Poorest (Q1)	2.05 (1.62–2.59)	<0.001	3.79	0.17 (0.14–0.2)	<0.001	4.21
	Poorer (Q2)	2.00 (1.61–2.48)	<0.001	2.98	0.27 (0.23–0.31)	<0.001	3.13
	Middle (Q3)	2.05 (1.62–2.59)	<0.001	3.79	0.17 (0.14–0.2)	<0.001	4.21
	Richer (Q4)	2.00 (1.61–2.48)	<0.001	2.98	0.27 (0.23–0.31)	<0.001	3.13
	Richest (Q5)	Ref			Ref		
**Ecological Zone**							
	Mountain	Ref			Ref		
	Hill	0.97 (0.80–1.17)	0.75	4.28	0.96 (0.8–1.16)	0.69	4.97
	Terai	1.45 (1.16–1.82)	<0.001	4.88	0.82 (0.67–1.01)	<0.05	4.53
**Residence**							
	Urban	Ref			Ref		
	Rural	1.03 (0.92–1.14)	0.36	1.85	0.95 (0.86–1.04)	0.25	1.91
**Provinces**							
	Province-1	Ref					
	Province-2	1.73 (1.45–2.07)	<0.001	2.58	0.65 (0.55–0.76)	<0.001	2.58
	Province-3	0.75 (0.60–0.94)	<0.01	1.74	1.14 (0.97–1.34)	0.12	1.74
	Province-4	0.73 (0.58–0.91)	<0.01	1.83	1.28 (1.09–1.51)	<0.001	1.83
	Province-5	1.20 (0.99–1.44)	<0.05	1.99	0.91 (0.78–1.06)	0.22	1.99
	Province-6	1.33 (1.09–1.63)	<0.01	2.07	0.66 (0.55–0.8)	<0.001	2.07
	Province-7	1.41 (1.18–1.68)	<0.001	1.90	0.57 (0.48–0.67)	<0.001	1.90
Observation (N)		9,519	11,158				
Hosmer-Lemeshow chi2(18)		19.82 (0.346)			19.42 (0.366)		
Mean VIF (Max)		2.75 (4.88)			2.85 (4.95)		
LR Chi2 (2)		577.95 (<0.001)			1591.88 (<0.001)		
Area under ROC curve		0.69			0.67		

### Factors associated with overweight and obesity

Being overweight or obese was significantly associated with sex and age of the participants (**Table 3**). The younger age adults (36–45 years) (aOR 3.05, 95% CI: 2.61–3.57, p<0.001) and females (aOR: 1.53, 95% CI: 1.39–1.68, p<0.001) were either overweight or obese, compared to those in other age groups and males, respectively. Similarly, in general, adults in all age groups were also more likely to be overweight or obese. In contrast, the adults who never married (aOR: 0.45, 95% CI: 0.40–0.58, p<0.001), had no education or preschool education only (aOR: 0.66, 95% CI: 0.56–0.78, p<0.001), and those in all wealth quintiles were less likely to have overweight or obesity. Interestingly, there was no difference in terms of overweight or obesity patterns by ecological regions and place of residence (urban vs. rural) (aOR: 0.95, 95% CI: 0.86–1.04, p = 0.25).

## Discussion

To the best of our knowledge, this is a first study ever been conducted to report the prevalence of underweight and overweight/obesity, using Asian specific BMI cut-offs [20, 24]. This is determined by measurement of height and weight and includes a nationally representative sample of Nepalese adults aged 18 years and older. Use of Asian specific cut-offs have been recommended by the WHO expert consultation based on the risk factors and morbidities patterns among Asian population [24–26]. These cut-off points define underweight ≤18.49 kg/m^2^, normal weight 18.5–22.99 kg/m^2^, overweight 23.0–27.49 kg/m^2^ and obesity ≥ 27.5 kg/m^2^, which are lower than WHO recommended criteria. This study uses the data available from the recently conducted nationwide survey of NDHS, 2016 [19].

The overall prevalence of overweight/obesity (29.35%) and underweight (17.24%) among both males and females is high. Compared with males, females are more likely to be both underweight (18.30%) and overweight (32.9%). The results demonstrate the co-existence of dual burden of underweight and overweight in both males and females. These findings are consistent with data from South Asian neighboring countries [27–29]. For example, a Bangladeshi study reported that 36% of adult women and 29.1% men where underweight and 24.4% of women and 20.5% of men were overweight or obese [27]. Similarly, another study conducted in Bangladesh (women underweight 24%, overweight 13% and obesity 3%) [28] and in Pakistan (women underweight 30%, pre-overweight 15%, overweight 25% and obesity 14%) [29] also reported the similar patterns of underweight and overweight/ obesity among the adults. These all studies conducted in neighboring countries used Asian cut-offs for calculating BMI [27–29]. A 2013 Nepal NCD risk factor study reported that 21.8% women and 21.0% men were overweight, which is less than the one reported in our study [9]. In India, among the Asian Indian Chennai population, the age standardized prevalence of obesity among the females was 47.4% and males 43.2% [30]. This is similar to our findings and also used Asian cut-offs to calculate BMI. Most of the studies conducted in countries of Asia used Asian cut-offs to categorize underweight, normal and overweight/ obesity, except a STEPS survey in Nepal [9], which used WHO global BMI cut-points, which categorizes underweight ≤18.49 kg/m^2^, normal weight 18.5–24.99 kg/m^2^, overweight 25.0–29.99 kg/m^2^ and obesity ≥ 30.0 kg/m^2^ [31].

There was upward u-shaped trend prevalence of overweight/ obesity, with adults (26 to 55 years) almost twice as likely to be overweight/obese compared to other age groups. However, we found little difference in terms of overweight/obesity in females compared to males (32.9% vs. 28.8%). The evidence base including the one from the LMICs also shows that more females are overweight and obese compared to their male counterparts [27, 32–34]. On the other hand, a study in African country of Botswana reported that 19.5% of males and 10.1% of females were underweight [35]. Compared to the developing countries, the prevalence of obesity among the women is higher in developed countries while male and female ratio to overweight is almost the same [36, 37].

We reported no significant difference in terms of prevalence of overweight or obesity in people residing in urban or rural areas. However, other studies in the past in Nepal [9] as well as neighboring countries including Bangladesh [27, 38], Myanmar [39] and India [40, 41], have shown that urban residents have higher prevalence of overweight and obesity compared to their rural counterparts. In these Asian countries, overweight or obesity is more common among the people with high education level and high income or wealth index [41–44], and this pattern is consistent to the findings presented in this study. There are several reasons to such scenario, including rapid and disorganized urbanization, increasing sedentary lifestyles, easy access to and consumption of unhealthy food and high energy drinks etc. Low BMI is often associated with low nutritional status and adverse health outcomes [45]. Previous studies suggested that the underweight in women of childbearing age is a risk factor for adverse pregnancy outcomes, such as intrauterine growth retardation or low-birth weight infants [46, 47]. Besides, being overweight/ obese is associated with increased risks of developing chronic NCD conditions [17, 18, 45].

As the chronic NCDs have become a major public health threat for Nepal, the healthcare system is not yet prepared to mitigate this growing burden of NCDs [48, 49]. Addressing this growing threat would require a multi-faceted approach and collaboration among professionals and institutions that have traditionally worked separately. Ensuring an equitable supply of primary health care services to the disadvantaged or underserved populations is a great challenge for governments of low- and middle-income countries in the Asia Pacific, particularly for the populations residing in remote or rural locations. The scarcity of healthcare facilities, lack of trained medical professionals (i.e. doctors, nurses) and long distance between the community and the nearest health facility underscore the need for alternate models for service delivery to reach each sector of the public with necessary health services and affordable medications. While the problem of NCDs in LMICs including Nepal is increasing rapidly, several studies have highlighted the importance of building health systems that primarily emphasizes community-based intervention approaches and uses locally available resources [50–52].

The strength of this study is that it is the analysis of a large nationally representative samples comprising both urban and rural adult populations in Nepal. However, we note few limitations. Since it was a cross-sectional study, we could not elucidate causality between nutritional status and its’ determinants, primarily the lifestyle and related factors. In addition, the NDHS 2016 did not include information on dietary habits, alcohol intake or physical activity and hence major determinants of nutritional status could not be explored.

## Conclusion

The findings presented in this study indicate co-existence of the double burden of underweight and overweight/obesity among Nepalese adults aged 18 years and above. The proportion of overweight and obesity is substantially high among the wealthiest, educated, and women adults. This indicates that the problem of overweight/obesity, is likely to worsen if no effective intervention strategies are developed and implemented. Nonetheless, underweight among adults still remains a major public health challenge among poor and uneducated population of LMICs. Both conditions are associated with increased risk of NCD morbidity and deaths due to NCD and related conditions. Therefore, effective public health intervention approaches to address these conditions and associated risk factors are essential. These could involve improved primary health care systems that emphasizes NCDs prevention and care, enhanced NCD awareness among general population, improved healthy lifestyle and use of multi-faceted approach and multi-sectoral collaboration in the efforts of prevention and control of NCDs and associated risk factors.
